# Experimental evidence of a pathogen invasion threshold

**DOI:** 10.1098/rsos.171975

**Published:** 2018-01-24

**Authors:** Tad A. Dallas, Martin Krkošek, John M. Drake

**Affiliations:** 1 Department of Environmental Science and Policy, University of California, Davis, CA, USA; 2 Odum School of Ecology, University of Georgia, Athens, GA, USA; 3 Department of Ecology and Evolutionary Biology, University of Toronto, Toronto, Ontario, Canada; 4 Center for the Ecology of Infectious Disease, University of Georgia, Athens, GA, USA

**Keywords:** epidemic, *Metschnikowia*, disease ecology, environmental transmission, pathogen emergence

## Abstract

Host density thresholds to pathogen invasion separate regions of parameter space corresponding to endemic and disease-free states. The host density threshold is a central concept in theoretical epidemiology and a common target of human and wildlife disease control programmes, but there is mixed evidence supporting the existence of thresholds, especially in wildlife populations or for pathogens with complex transmission modes (e.g. environmental transmission). Here, we demonstrate the existence of a host density threshold for an environmentally transmitted pathogen by combining an epidemiological model with a microcosm experiment. Experimental epidemics consisted of replicate populations of naive crustacean zooplankton (*Daphnia dentifera*) hosts across a range of host densities (20–640 hosts l^−1^) that were exposed to an environmentally transmitted fungal pathogen (*Metschnikowia bicuspidata*). Epidemiological model simulations, parametrized independently of the experiment, qualitatively predicted experimental pathogen invasion thresholds. Variability in parameter estimates did not strongly influence outcomes, though systematic changes to key parameters have the potential to shift pathogen invasion thresholds. In summary, we provide one of the first clear experimental demonstrations of pathogen invasion thresholds in a replicated experimental system, and provide evidence that such thresholds may be predictable using independently constructed epidemiological models.

## 1. Introduction

Central to the study of infectious disease dynamics is the concept of a critical threshold to pathogen invasion as a function of host density (i.e. a pathogen invasion threshold), below which a pathogen is unable to invade a host population [1,2]. This critical threshold is a commonly sought target for horticultural [1], wildlife [3] and human [4] disease control. The importance of host density to the emergence and spread of infectious disease has been demonstrated in many disease systems [5–7], but may be strongly influenced by environmental factors [8]. Apart from the influence of fluctuating environmental conditions, pathogen invasion thresholds may be difficult to measure in field populations as a result of data scarcity, lack of replicated experimentation and the effects of host ecology (e.g. behaviour, social structure) [9].

Despite limited evidence for these thresholds in field populations, there are numerous theoretical studies identifying critical thresholds for pathogen invasion using epidemiological models [10–12]. This has resulted in a body of theory and indirect evidence for invasion thresholds without direct experimental evidence. In epidemiology, in particular, the basic reproduction number (*R*_0_) is a fundamental quantity that formalizes the threshold concept, providing a boundary between pathogen invasion (*R*_0_>1) and pathogen extinction (*R*_0_<1). Given that *R*_0_ is a common target for vaccination and pathogen control efforts in both theoretical [13,14] and applied [15,16] contexts, it may seem counterintuitive that pathogen invasion thresholds have largely been examined in theoretical studies. However, the estimation of *R*_0_ requires an epidemiological model capable of capturing host and pathogen dynamics, which is a challenging task for wildlife pathogens, or host–pathogen interactions occurring in fluctuating environments, as fluctuating environments can change infectious period, host susceptibility or host contact patterns.

This challenge may be responsible for the limited support for pathogen invasion thresholds in wildlife populations [9,17–19]. A necessary condition for a pathogen invasion threshold is the dependence of the rate of pathogen transmission on host density, such that invasion thresholds are theoretically predicted to be absent from systems with frequency-dependent transmission [20]. For environmental and density-mediated pathogen transmission, the existence of the pathogen invasion threshold can occur simply by high host density enhancing the probability that a host contacts an infected host or an environmental pathogen spore [21]. Further, changes to behaviour or host life history as a function of density could influence invasion thresholds. For instance, transmission of an environmentally transmitted pathogen could exhibit a threshold if hosts alter their contact patterns or feeding behaviour as a function of conspecific density. Despite the inherent difficulty in examining pathogen invasion thresholds in natural systems, the generation of a body of epidemiological theory concerning pathogen invasion without experimental demonstration creates a clear knowledge gap.

We provide experimental evidence of a pathogen invasion threshold, using an experimental system of a *Daphnia* host species infected by an environmentally transmitted fungal parasite. Through epidemiological modelling we further examine how parameter uncertainty can influence pathogen invasion probability, providing insight into the robustness of pathogen invasion threshold estimates. We also provide a test of the idea of the upper host density threshold [22], in which high host density results in suppressed feeding and pathogen transmission, leading to reduced pathogen transmission and subsequent pathogen invasion probability. Lastly, we examine how gradients of key epidemiological parameters can influence pathogen invasion thresholds, as many relevant parameters are subject to environmental conditions. These results demonstrate the existence of a critical host density for the invasion of an environmentally transmitted pathogen consistent with theoretical predictions, and examine how sensitive pathogen invasion thresholds may be to changes in several important parameters.

## 2. Material and methods

### 2.1. Host–pathogen system

We examined a model host–pathogen system consisting of a freshwater cladoceran (*Daphnia dentifera*) parasitized by an environmentally transmitted fungal pathogen (*Metschnikowia bicuspidata*). This experimental system offers the ability to control for genetic effects (as *Daphnia* are parthenogenetic) and to examine highly replicated pathogen challenges across a gradient of initial population sizes. Previous studies of *Daphnia*–microparasite interactions have characterized many important demographic processes [23–28], providing information on variation in key epidemiological parameters.

The host reproduces parthenogenetically in favourable environments, and typically produces a clutch approximately every 3–6 days after maturation, with clutch sizes of up to 20 individuals [29]. The pathogen is transmitted during host filter-feeding, piercing the gut wall and growing inside infected hosts. During the time when the pathogen grows in an infected host, there is no pathogen shedding into the environment or host-to-host transmission. Infected hosts experience decreased fecundity, and parasite-induced mortality occurs within approximately 14 days after transmission [29]. As there is no direct (host-to-host) transmission, exposure of susceptible hosts to environmental pathogen in the environment is a necessary step in the transmission process. Infected hosts release spores into the environment upon death, which contribute to the next wave of infection when they are consumed by susceptible host individuals.

### 2.2. Experimental design

Populations of non-gravid adult *D. dentifera* hosts were established in 50 ml of pond water media (25% pond water, 75% deionized water) along a geometric series of initial host densities (20, 40, 80, 160, 320 and 640 hosts l^−1^), corresponding to host abundances of 1, 2, 4, 8, 16 and 32 hosts per microcosm. Each population (*n*=15 per treatment; *n*=90 populations total) was fed 2 mg algal dry weight l^−1^ of a suspension of freeze-dried pulverized *Spirulina* sp. in deionized water daily (as performed previously [30,31]). This food concentration was constant across treatments, regardless of host density, as resource density may have a more pronounced effect on host filtering rate and subsequent pathogen transmission [32] than *per capita* resource levels, and feeding populations proportional to host abundance would be logistically infeasible. Since censusing occurred twice per week and feeding occurred daily, a dynamic feeding regime would be too temporally coarse, potentially leading to the introduction of further variation in population sizes and filtering rates. The resource levels provided were capable of sustaining all host densities examined, as supported by the density of individuals reaching nearly 2000 individuals l^−1^, or 100 individuals in a single 50 ml test tube. Each population was inoculated with approximately 500 pathogen spores (10 spores ml^−1^) based on previous studies [27].

Populations were censused every 3–4 days for the course of experimental epidemics. During censusing, individuals were exposed to the lowest microscope light setting, and kept in media to maximize survivorship. Dead hosts were not removed, as they may still contain small numbers of spores, and removal might have affected infection dynamics. Individuals were then placed back in their original test tubes.

The observation of one infected individual could occur in our experiment without resulting in pathogen persistence (endemic or epidemic). To examine this, we calculated the fraction of populations containing primary infections and secondary infections, in addition to epidemic-scale statistics (epidemic size and maximum infection prevalence) to provide a comparison between initial infection events and longer-term infection dynamics. Primary infection events were defined as those occurring on or before day 11, which captures only infection events from the initial spore dose, and not from pathogen spread through an infected host. Secondary infection captures the subsequent infection of individuals after the primary infection event(s), and was defined as infection events occurring on or after day 21. Epidemic size was defined as the area under the curve of infection prevalence over time for each population, and maximum infection prevalence was the highest observed infection prevalence throughout the course of each epidemic.

### 2.3. Epidemiological model

We complemented our experimental epidemics with a susceptible-infected-pathogen model to investigate the existence of a host density threshold. This model, parametrized independently of experimental epidemics, was used to obtain quantitative predictions of pathogen invasion thresholds in the *Daphnia*–microparasite system that may scale to other environmentally transmitted pathogens.

2.1 $$\begin{array}{r} \dot{S\;}\;=b(N)S+\phi b(N)I-d_{0}S-u\gamma SP, \end{array}$$

2.2 $$\begin{array}{r} \dot{I\;}\;=u\gamma SP-I(d_{0}+v) \end{array}$$

2.3 $$\begin{array}{rl} \;\mathrm{and}\;\dot{P\;}\; & =I\theta (d_{0}+v)-\mu P. \end{array}$$

In our model, individuals reproduce and die according to density-dependent fecundity (*b*(*N*)=*b*_0_−*b*_1_*N*) and density-independent mortality (*d*_0_), where the overall density of individuals *N* is equal to the susceptible individuals *S* plus the infected individuals *I*. Susceptible individuals give birth and die according to these fecundity and mortality functions, where *b*_0_ and *d*_0_ represent the maximum birth and minimum death rates, respectively. Reduced fecundity at high host density constrains the population dynamics according to a logistic fashion, where the carrying capacity is given by (*b*_0_−*d*_0_)/*b*_1_.

Infected individuals also contribute to the birth of susceptible host individuals (i.e. there is no vertical transmission), discounted by a constant *ϕ*. Susceptible host individuals become infected as a function of susceptible (*S*; equation 2.1) and pathogen (*P*; equation 2.3) populations, susceptible host individual feeding rate (*γ*) and environmental pathogen spore infectivity (*u*). These two parameters (*γ* and *u*) make up the transmission term. Infected individuals contribute *θ* pathogen spores to the environmental pathogen population *P* after density-independent mortality, which is the sum of the baseline mortality (*d*_0_) and a disease-induced mortality (i.e. virulence; *v*). Environmental pathogen spores become uninfective according to the pathogen mortality rate *μ*, and this was the only cause of spore mortality. Parameter definitions and estimates are provided in table 1.

**Table 1.** Parameters used in our epidemiological model. Ranges are given for key infection parameters (*γ*, *u* and *θ*) sampled to incorporate parameter uncertainty into model-predicted pathogen invasion thresholds.

parameter	units	definition	value	references
*b*_0_	day^−1^	host birth rate	0.45	[31],[33]
*d*_0_	day^−1^	host death rate	0.15	[31]
*b*_1_	day^−1^	host birth rate	4.7×10^−4^	—
*ϕ*	—	fecundity reduction by infection	0.75	[26]
*u*		per spore infectivity	0.0005–0.005	[22],[34]
*γ*	l ind^−1^ day^−1^	host filtering rate	0.001–0.01	[35],[36]
*θ*	number	mean spore load per infected host	5×10^3^–1.5×10^4^	[37],[25]
*v*	day^−1^	pathogen induced host mortality	0.05	[25]
*μ*	day^−1^	death rate of environmental pathogen	0.75	[29]

The environmental pathogen population (equation 2.3) is the sum of inputs from dead infected hosts, and losses from environmental degradation of spores at a rate of *μ*. A slightly more complicated model would incorporate loss of environmental pathogen spores from host foraging, as previous studies have suggested that this could be important [22]. However, for the sake of simplicity, we present the model without spore loss through host foraging here, and provide analyses of a model incorporating host foraging effects in the electronic supplementary material. The results are strikingly similar, and the simplified model avoids complications concerning the details of host foraging, such as the effect of gut passage on pathogen viability, intraspecific variation in foraging rates, and the dependence of foraging rate on conspecific density, resource levels or pathogen density.

We estimated *R*_0_ as the product of the total pathogen produced by a single infected host (i.e. *θ*), the total number of spores consumed by hosts during the invasion window and average environmental spore lifespan (i.e. *μ*^−1^). The resulting formula (equation 2.4) is identical to *R*_0_ determined using the next generation approach (see electronic supplementary material). Here, S* is the only part of *R*_0_ that is directly related to host density. We define S* as the mean population size observed in the transient window after pathogen exposure and before primary infection was assessed (day 11) in the experimental epidemics, providing a link between the transient dynamics observed in experimental infections and the estimation of pathogen invasion thresholds from our epidemiological modelling effort.

2.4 $$R_{0}=\theta \gamma uS^{*}\frac{1}{\mu }=\frac{\theta \gamma uS^{*}}{\mu }.$$

### 2.4. Allowing variability in parameter estimates

It can be difficult to accurately estimate many epidemiological parameters, and this uncertainty can influence model predictions. We used parameter estimates from published research (table 1) to determine a range of critical threshold values. To explore model behaviour in parameter space, we sampled values of host filtering rate (*γ*), per spore infectivity (*u*) and the number of spores produced per infected host (*θ*) from uniform distributions, bounded by empirical parameter estimates, except for *u*, for which few data were available (table 1). These three parameters are important to pathogen transmission, as *θ* determines how much pathogen is present, and *γ* and *u* together determine the pathogen transmission rate. Previous work has emphasized that *θ*, *u* and *γ* can strongly influence epidemic dynamics [24,38], and are sensitive to resource concentrations and environmental influences. Distributions of *θ*, *u* and *γ* were sampled 1000 times to obtain a set of possible parameters, while other values corresponding to host demography (*b*_0_, *b*_1_, *d*_0_, *ϕ*), disease-induced mortality (*v*) and pathogen demographics (*μ*) (table 1) were treated as constants here.

### 2.5. Model simulations

These parameter combinations were used to characterize variability in pathogen invasion both in terms of *R*_0_ and the probability of pathogen invasion as predicted by deterministic and stochastic model simulations. The stochastic model was used to examine the influence of demographic stochasticity on model outcomes, while the study of parameter space was a way to examine the influence of environmental variation. State transitions in the stochastic model (e.g. susceptible host to infected host) were stochastic, except for the loss of pathogen from the environment (*μ*), which we modelled as a deterministic process. Stochastic simulations were computed using the next reaction method [2,39] implemented in the adaptivetau R package [40]. Deterministic and stochastic realizations of the model were simulated for all sampled parameter combinations (as defined above) for 11 days, which corresponds to the invasion window examined in our experimental epidemics.

The probability of pathogen invasion was based on the fraction of parameter combinations that resulted in at least one infected individual in deterministic and stochastic simulations. Confidence intervals were obtained from the binomial distribution, as pathogen invasion is a binary outcome. Pathogen invasion probability was quantified in the same manner for model simulations and experimental epidemics; the minimum criteria for pathogen invasion in this system is the infection of one individual (i.e. a completion of half the life cycle of the environmentally transmitted parasite). This is because the amount of pathogen spores used to initiate the experiment was more than an order of magnitude less than the number of pathogen spores an infected host produces. Thus, the infection of one individual would suggest that *R*_0_>1, as the pathogen death rate cannot offset the contribution of pathogen spores produced by an infected host in the pathogen transmission term (*uγSP*). This provides a clear link between invasion probability as defined by either the observation of one infected individual or *R*_0_>1. For model simulations, pathogen invasion was defined both as *R*_0_>1 and the observation of a single infection event within the pathogen invasion window considering parameter uncertainty as described above. This is the same invasion criterion we use for primary infection events in our experimental epidemics. Further, we obtained an analytical expression for the invasion threshold (one individual becoming infected before day 11) for the stochastic model (see electronic supplementary material).

### 2.6. Invasion threshold sensitivity

Understanding which parameters influence pathogen invasion thresholds is important, as many of these parameters will change with environmental conditions [41,42] and population genetics [43]. For instance, previous work has demonstrated that nitrate can influence pathogen spore mortality (*μ*) [31]. The obvious parameters that will have strong effects on pathogen invasion thresholds are those that influence pathogen transmission and loss of pathogen from the environment (i.e. two key aspects of *R*_0_). To understand how specific parameters influence pathogen invasion thresholds, we examined model-predicted pathogen invasion thresholds along gradients of three key parameters present in *R*_0_, which correspond to pathogen transmission (*γ* and *u*) and pathogen mortality (*μ*). We estimated pathogen invasion probability based on the analytical solution of the stochastic model. This potentially underestimates invasion probability at high host densities. We provide a further study of how variability in these key parameters influenced *R*_0_ across the gradient of host densities in the electronic supplementary material.

## 3. Results

### 3.1. Invasion thresholds in model and experiment

**Figure 1.**
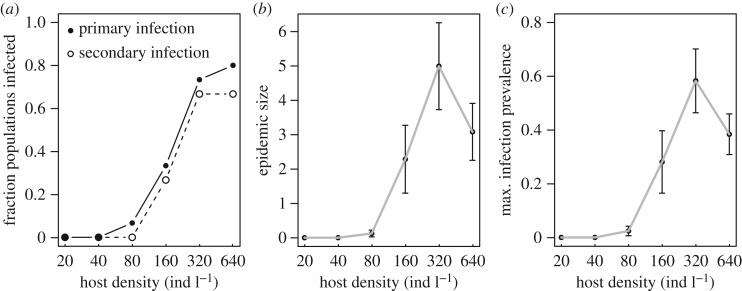
Initial host density (*x*-axis) strongly influenced infection dynamics and pathogen invasion in experimental epidemics, evidenced by the fraction of primary and secondary infections (*a*), epidemic size (area under the infection curve; *b*) and maximum infection prevalence (*c*).

**Figure 2.**
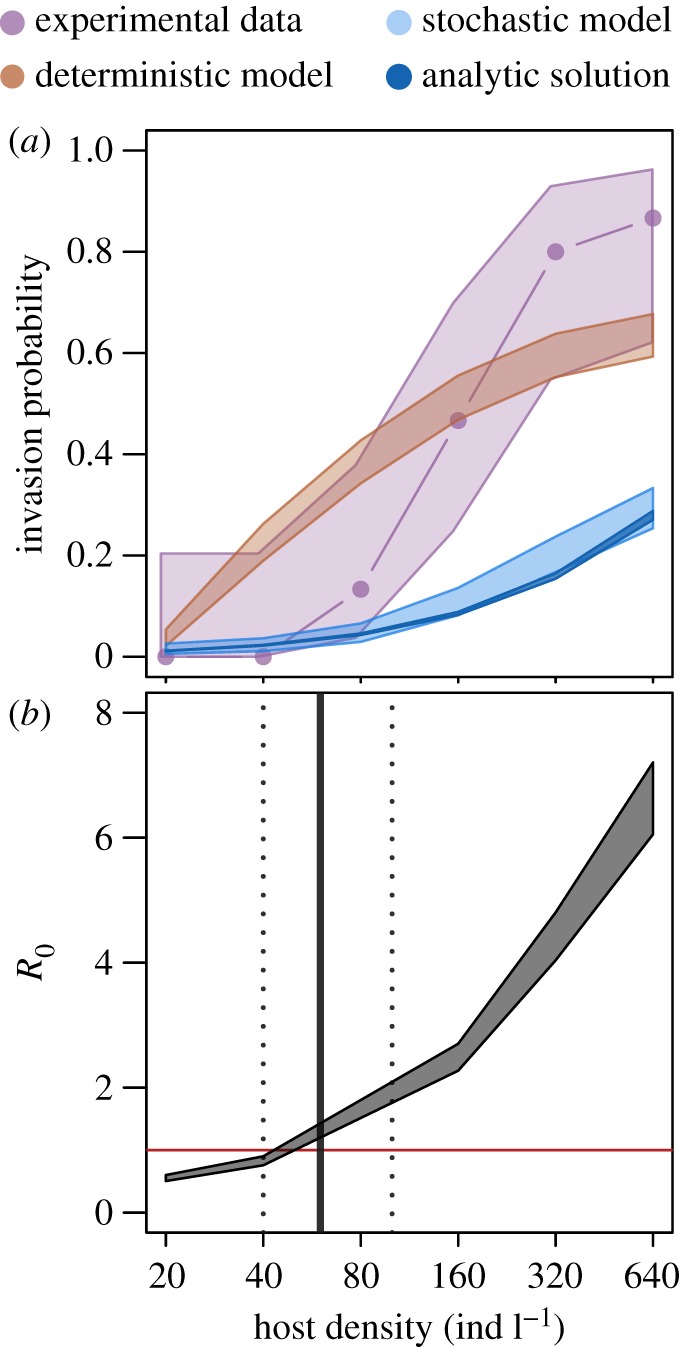
The probability of pathogen invasion (*a*) from deterministic and stochastic simulations compared to data from experimental epidemics. The darker blue shaded region in the upper panel corresponds to the analytical solution of the pathogen invasion probability for the stochastic model. Shaded regions correspond to binomial confidence intervals from model simulations, which sampled parameter values for *u*, *γ* and *θ*. From these model simulations, we calculated *R*_0_ (*b*). Vertical grey line corresponds to the host density at which 50% of parameter combinations result in *R*_0_>1, with corresponding dashed lines corresponding to 35% and 65% parameter combinations resulting in *R*_0_>1. The horizontal red line indicates *R*_0_=1.

A clear pathogen invasion threshold was observed in experimental trials, with no successful pathogen transmission at the two lowest density treatments and a sudden increase in infection events between 80 and 160 host individuals l^−1^ (figure 1). We found strikingly similar patterns between invasion-scale and epidemic-scale patterns, suggesting that our measure of pathogen invasion within the invasion window is correctly identifying host density thresholds to pathogen invasion. Only a handful of instances occurred in which an initial infection did not lead to subsequent pathogen invasion, with these instances corresponding to host population crashes. Pathogen invasion thresholds—defined either as *R*_0_>1 or as the probability of observing one infected individual following pathogen exposure—predicted by model simulations were qualitatively similar to the observed pathogen invasion probability from experimental epidemics (figure 2).

Specifically, the predicted pathogen invasion thresholds from deterministic and stochastic simulations largely overlapped with the binomial confidence intervals from experimental epidemics, in which the experimentally determined pathogen invasion probability appears to be a combination of deterministic and stochastic dynamics. That is, the stochastic model more closely matched experimental populations at low host densities, owing to the ability of the model to capture demographic stochasticity, while the deterministic model was able to better match experimental populations at high host densities (figure 2).

One interesting idea proposed by Civitello *et al.* [22] is that if hosts suppress their feeding at high initial densities, epidemic size (quantified as area under the epidemic curve) could be reduced at these densities; this could potentially result in an upper host density threshold to pathogen invasion (discussed further in the electronic supplementary material). However, we found little support for the existence of this upper host density threshold, as the fraction of populations with at least one infected individual recorded saturated, but did not decrease, at high host densities (figure 1).

### 3.2. Variability in parameter estimates

Confidence ranges for both the deterministic and stochastic simulations were obtained by sampling parameter space for three key parameters—host filtering rate (*γ*), per spore infectivity (*u*) and the number of spores per infected host (*θ*)—and simulating dynamics. Binomial confidence intervals calculated from pathogen invasion success from deterministic and stochastic simulations which sampled the parameter space provide evidence that pathogen invasion probability is relatively robust to variability in parameter estimates (figure 2).

### 3.3. Invasion threshold sensitivity

**Figure 3.**
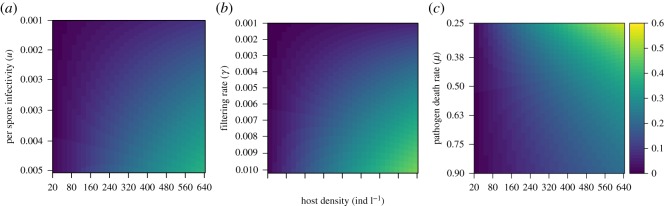
The probability of pathogen invasion (colour legend) as a function of per spore infectivity (*u*; *a*), host filtering rate (*γ*; *b*) and environmental pathogen death rate (*μ*; *c*).

While the incorporation of parameter variability did not strongly influence invasion probability, examining gradients in individual parameters while holding other parameters constant revealed a strong influence of host filtering rate (*γ*), per spore infectivity (*u*) and pathogen death rate (*μ*) on pathogen invasion thresholds. This suggests that pathogen invasion probabilities estimated from the analytical solution of the stochastic model were sensitive to variation in these key parameters (figure 3). Qualitatively, changes to pathogen death rate (*μ*) had less of an impact on the probability of pathogen invasion relative to pathogen transmission terms (*u* and *γ*), suggesting that changes to environmental pathogen survival may not influence pathogen invasion nearly as much as changes to parameters related to pathogen encounter and transmission, such as host foraging rate or pathogen infectivity. While the stochastic model, and associated formula for the pathogen invasion probability, underestimates pathogen invasion probability at high initial densities, the rate of change is similar to observe how shifts in parameters could strongly influence pathogen invasion thresholds.

## 4. Discussion

Combining replicated experimental infection trials with an epidemiological model, we have provided an experimental demonstration and theoretical justification for the existence of the pathogen invasion threshold for an environmentally transmitted pathogen. Predictions for the pathogen invasion threshold—defined as both *R*_0_>1 and as the probability of observing a primary infection event—obtained from deterministic and stochastic realizations of our epidemiological model were qualitatively similar to experimental epidemics, suggesting that the invasion threshold may be estimated from a simple independently parameterized epidemiological model. However, we found no evidence for an upper threshold to pathogen invasion, as hypothesized in earlier studies [22]. Pathogen invasion thresholds may depend on environmental context, which we incorporated by allowing uncertainty in several key parameters (*γ*), *u*, *θ* and *μ* (see electronic supplementary material) and further examining the probability of pathogen invasion along gradients of host filtering rate (*γ*), per spore infectivity (*u*) and pathogen death rate (*μ*). The range of pathogen invasion thresholds obtained by exploring this parameter space suggests that small amounts of variability will probably not strongly influence pathogen invasion probability (figure 2), but that small directional changes to key infection parameters can exert stronger influence on pathogen invasion (figure 3). Together, these findings provide empirical evidence for the existence of a critical threshold to pathogen invasion in an environmentally transmitted pathogen, showing consistency with both deterministic and stochastic model simulations that capture important features of super-critical pathogen transmission.

Understanding the factors contributing to pathogen emergence remains a key goal of disease ecology and epidemiology. While previous studies have examined pathogen invasion thresholds of directly transmitted pathogens [44], invasion thresholds of environmentally transmitted pathogens are less well understood [45–47]. Notably, while much theory has been developed examining invasion thresholds for environmentally transmitted pathogens [1,46], very few experimental tests of this theory have been performed [48]. The *Daphnia* host–pathogen model described in this paper may be generalizable to other environmentally transmitted pathogen systems [21]. For instance, an extension of our model could consider a pathogen that reproduces in the environment, or that is able to be transmitted through both direct host contact and from an environmental source [49]. Some environmentally transmitted pathogens may have dynamics similar to directly transmitted pathogens if the pathogen is short-lived and has limited dispersal [21,50]. The host–pathogen system we examined is characteristic of this situation (see resulting waves of infection in electronic supplementary material, figure A4), in which transmission occurs in a small window immediately following host death, and environmental pathogen spores are relatively short-lived [31].

There were differences between stochastic and deterministic model realizations, and both of our models from the experimental data. That is, while invasion probabilities from the deterministic model largely overlapped the experimental data, the sigmoidal shape of the relationship in experimental data was not captured by deterministic or stochastic realizations. Further, demographic stochasticity increased the probability that the pathogen would not invade either through host extinction or the pathogen degrading in the environment before it could result in a successful infection. However, our calculation of *R*_0_ from parameters—measured independently of experimental trials—captured pathogen invasion in experimental trials quite well. Pathogen invasion thresholds may be more difficult to predict in natural populations where transmission may be environmentally dependent [27,31,51], creating a challenge for future research efforts to accurately capture transmission and infection dynamics in natural populations.

Our experimental demonstration of a core concept in epidemiology provides a platform to study how shifting environments, species interactions and pathogen pressure influence the pathogen invasion threshold. Evidence that invasion thresholds may shift as a function of environmental context is provided by the range of threshold values observed for the wide range of parameter space sampled in our model, including many important parameters related to pathogen transmission (*u* and *γ*), production of infectious spores (*θ*) and pathogen demography (*μ*). This generates a number of open questions concerning how environmental covariates, species interactions or genetic factors may influence this critical threshold. An understanding of how pathogen emergence may be influenced by environmental context is an important research area that may be informed through the use of controlled microcosm studies. Lastly, the incorporation of stochasticity into invasion threshold estimates, and the parametrization of models using existing data are both tools that could improve pathogen threshold estimation for wildlife and managed populations.

## Supplementary Material

Supplemental Material

## Data accessibility

Data and code from this study are available on figshare (https://doi.org/10.6084/m9.figshare.5133001).
