# Supplementary material for: Experimental evidence of a pathogen invasion threshold
Source: R Soc Open Sci. 2018 Jan 24;5(1):171975. doi: 10.1098/rsos.171975 (PMC5792953; doi:10.1098/rsos.171975)
Supplement: Supplemental Material [file rsos171975supp1.pdf]

Supplementary Materials for: Experimental evidence of a pathogen invasion threshold

Tad A. Dallas [1, 5, ‡]   Martin Krkošek [2]   John M. Drake[3,4 ‡]

Affiliations:

1. Department of Environmental Science and Policy, University of California, Davis, CA, USA
2. Department of Ecology and Evolutionary Biology, University of Toronto, Toronto, On, Canada
3. Odum School of Ecology, University of Georgia, Athens, GA,USA
4. Center for the Ecology of Infectious Disease, University of Georgia, Athens, GA, USA
5. Corresponding author: `tdallas@ucdavis.edu`

‡ ORCIDs: Dallas, 0000-0003-3328-9958; Drake, 0000-0003-4646-1235

Sensitivity of results to parameterization of carrying capacity

Our parameterization of carrying capacity, defined as $K = (b_0 - d_0)/b_1$ did not influence model predictions (Figure A1). We examined a broad range of carrying capacities, finding that the estimation of pathogen invasion probability was insensitive to changes in carrying capacity. The value of b_1 in the main text corresponds to a carrying capacity of 640 individuals L^{-1}, which was the maximum density we examined in our experimental infection trials. However, as we demonstrate here, our findings are robust to other values of b_1. This is not surprising, as the focus is on the transient period after pathogen exposure, such that carrying capacity does not play a large role in pathogen invasion.

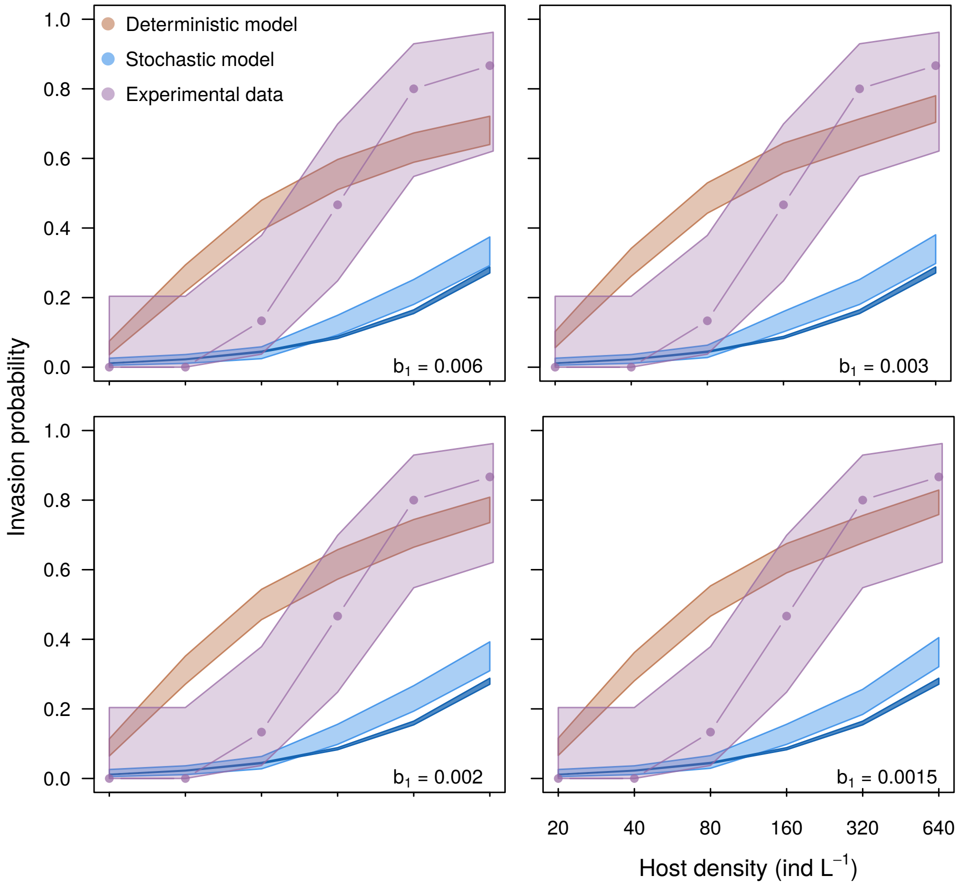

Figure A1: A recreation of main text Figure 2 except with carrying capacity (K) set to a) 1000, b) 2000, c) 3000, or d) 4000 individuals L^{-1}. For each plot, we plot the probability of pathogen invasion for deterministic (blue shaded region) and stochastic (green shaded region) model realizations compared to data from experimental epidemics (red points). Shaded regions represent 95% binomial confidence intervals for the full set of model runs incorporating parameter uncertainty in u, γ, and θ. The darker blue shaded region in the upper panel is the analytical solution of the pathogen invasion probability for the stochastic model.

Analytical derivation of invasion probability from stochastic model

In the stochastic version of our model, the rate at which i individuals become infected by the fungal pathogen is a non-homogeneous Poisson process. This is a result of environmental pathogen death altering the potential number of infection events over time. This derivation does not consider spore loss through host foraging, as this does not strongly influence pathogen invasion. Here, we can define the intensity function $\lambda(t)$ as

$$\lambda(t) \;=\; u\gamma P^{*} e^{-\mu t} \tag{1}$$

where the per spore infectivity (u) and host filter rate (γ) influence the rate at which a host ingests infective pathogen, and the environmental pathogen (P^{*}) is lost from the system at rate μ. The mean intensity over the experimental time period of pathogen invasion (0 - 11 days) then becomes

$$\Lambda(t_0, t_{11}) \;=\; \int_0^{11} \lambda(t)\,\mathrm{d}t \tag{2}$$

The probability of observing n infection events in this time period is then

$$P(n) \;=\; \frac{\Lambda^n}{n!}\, e^{-n} \tag{3}$$

and the probability of observing no infection events ($n = 0$), and the probability of observing one or more infection event (i.e., pathogen invasion) are

$$\begin{aligned} P(n=0) \;&=\; (e^{-\Lambda})^x \\ P(n \geq 1) \;&=\; 1 - (e^{-\Lambda})^x \end{aligned}$$

where x is the number of susceptible hosts in the population.

Sensitivity of R_0 to parameter uncertainty

The four parameters involved in the calculation of R_0 (γ, θ, u, and μ) differed in the way they influence the relationship between host density and R_0, both in terms of the general shape of the relationship and the size of the standard error bars (Figure A2). For instance, the number of spores produced per infected host θ did not influence R_0 as strongly as pathogen death rate (μ), spore consumption rate (u), or per spore infectivity (γ). This is potentially due to the latter three parameters contributing to the infection process (i.e., the transition from environmental pathogen to infected host), while θ determines the transition from infected host to the amount of environmental pathogen for the next round of infection.

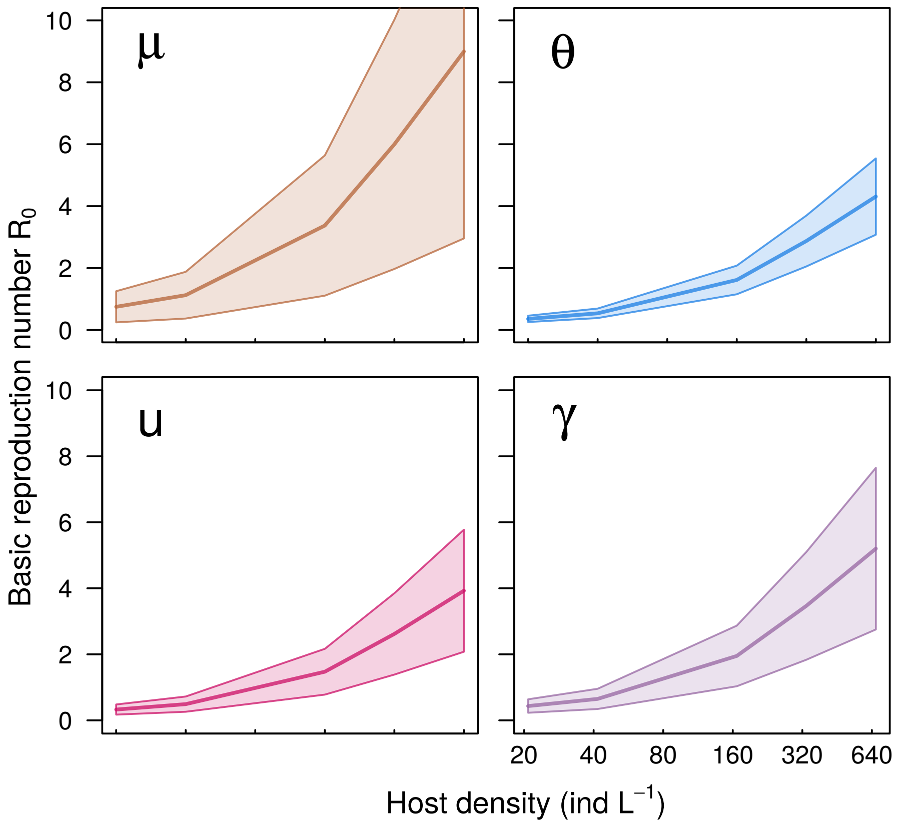

Figure A2: The effect of host density and parameter uncertainty on the basic reproduction number (R_0). The solid line is the mean R_0 and the standard error polygons are two times the standard error away from the mean for visibility.

Calculation of R_0 for the SI model with environmental pathogen

Below, we outline the structural forms of two SI models with environmental pathogen (P), differing in the rate at environmental pathogen loss as a function of host foraging. Host foraging had almost no influence on critical host density for pathogen invasion (Figure A3) except at large initial host densities. Further, it is unlikely that host foraging on pathogen could reduce infection dynamics, except at large densities, or very high feeding rates (γ). As crowding tends to reduce host filtering rate [1], it is unlikely to observe both a dense population and a high filtering rate. We provide R_0 calculations for both models, though the R_0 calculation for the SI model without spore loss through host foraging is also in the main text.

Model without spore loss through host foraging

$$\dot{S} = b(N)S + \phi b(N)I - d_0 S - u\gamma SP \tag{4}$$

$$\dot{I} = u\gamma SP - I(d_0 + v) \tag{5}$$

$$\dot{P} = I\theta(d_0 + v) - \mu P \tag{6}$$

Next generation matrix determination of R_0 We used the next generation matrix method [2]), which is the dominant eigenvalue of the product of two matrices (F and V^{-1}). The calculation of R_0 follows the "transition" scenario of [3], in which the pathogen does not grow outside of hosts, but essentially acts as a second state of infected host. Specifically, hosts contribute pathogen to the environment, and that environmental pathogen is the only source of infection for hosts (i.e., there is no host-host transmission).

$$\mathcal{M} = FV^{-1} = \begin{bmatrix} 0 & u\gamma S^* \\ 0 & 0 \end{bmatrix} \begin{bmatrix} (d_0 + v) & 0 \\ -(d_0 + v)\theta & \mu \end{bmatrix}^{-1} \tag{7}$$

$$max(eig(\mathcal{M})) = R_0 \; = \; \frac{\theta\gamma u S^*}{\mu} \tag{8}$$

Model with spore loss through host foraging

$$\dot{S} \; = b(N)S \; + \; \phi b(N)I \; - \; d_0 S \; - \; u\gamma SP \tag{9}$$

$$\dot{I} \; = \; u\gamma SP - I(d_0 + v) \tag{10}$$

$$\dot{P} \; = \; I\theta(d_0 + v) - \mu P - \gamma NP \tag{11}$$

Heuristic formulation of R_0 In the main text, we defined R_0 from first principles. The same method can be applied to the model incorporating spore loss through host foraging. From first principles, R_0 would correspond to the product of the total pathogen produced by a single infected host, the total number of spores consumed by hosts when $S = S^*$, and average environmental spore lifespan. This is the same formulation as in the model without host foraging on spores, but with a reduction in environmental spore lifespan proportional to the host foraging rate.

$$R_0 \; = \; (\theta)(\gamma u S^*)\left(\frac{1}{\mu + \gamma S^*}\right) = \frac{\theta\gamma u S^*}{\gamma S^* + \mu} \tag{12}$$

Next-generation R_0 calculation The calculation of R_0 using the next generation matrix technique is nearly equivalent for this model relative to the model without spore loss through host foraging, except that pathogen is lost from the V matrix not only through pathogen death (μ), but also through a foraging rate (γ) scaled by the average transient host density during the invasion window (S^*).

$$\mathcal{M} \; = \; FV^{-1} = \begin{bmatrix} 0 & u\gamma S^* \\ 0 & 0 \end{bmatrix} \begin{bmatrix} (d_0 + v) & 0 \\ -(d_0 + v)\theta & \mu + u\gamma S^* \end{bmatrix}^{-1} \tag{13}$$

$$max(eig(\mathcal{M})) = R_0 = \frac{\theta\gamma u S^*}{(\gamma S^*) + \mu} \tag{14}$$

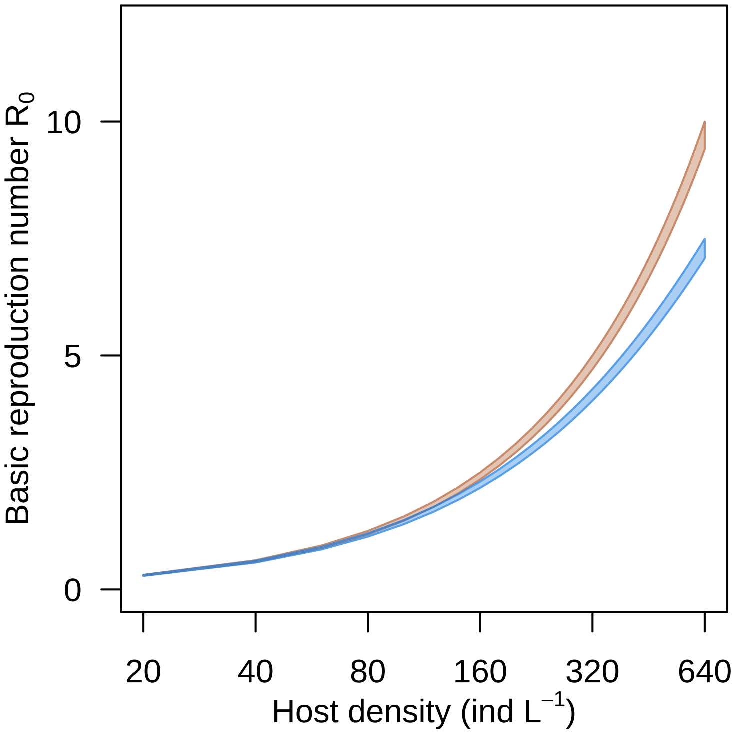

Figure A3: Model estimates of R_0 did not strongly differ when comparing models with (blue polygon) and without (orange polygon) spore loss through host foraging. Only at very high host densities would spore foraging be predicted to suppress R_0, and this suppression is not enough to influence pathogen invasion thresholds.

Possible existence of an upper host density threshold

Previous research has suggested that intraspecific interactions among hosts may result in feeding suppression, which could reduce epidemic size and perhaps even create an upper host density threshold to pathogen invasion at high host densities [4]. Further, this would result in a non-monotonic relationship between host density and the pathogen invasion threshold, as a result of suppressed host feeding at high host densities resulting in reduced pathogen transmission. We did not find support for an upper host density threshold to pathogen invasion (Figure A4 inset). On the other hand, epidemic size and max infection prevalence declined in the highest host density treatment, suggesting that epidemics weren't as severe when host density was extremely high. This suggests that dense host populations may experience smaller overall epidemics, but casts doubt on the existence of an upper host density threshold to pathogen invasion.

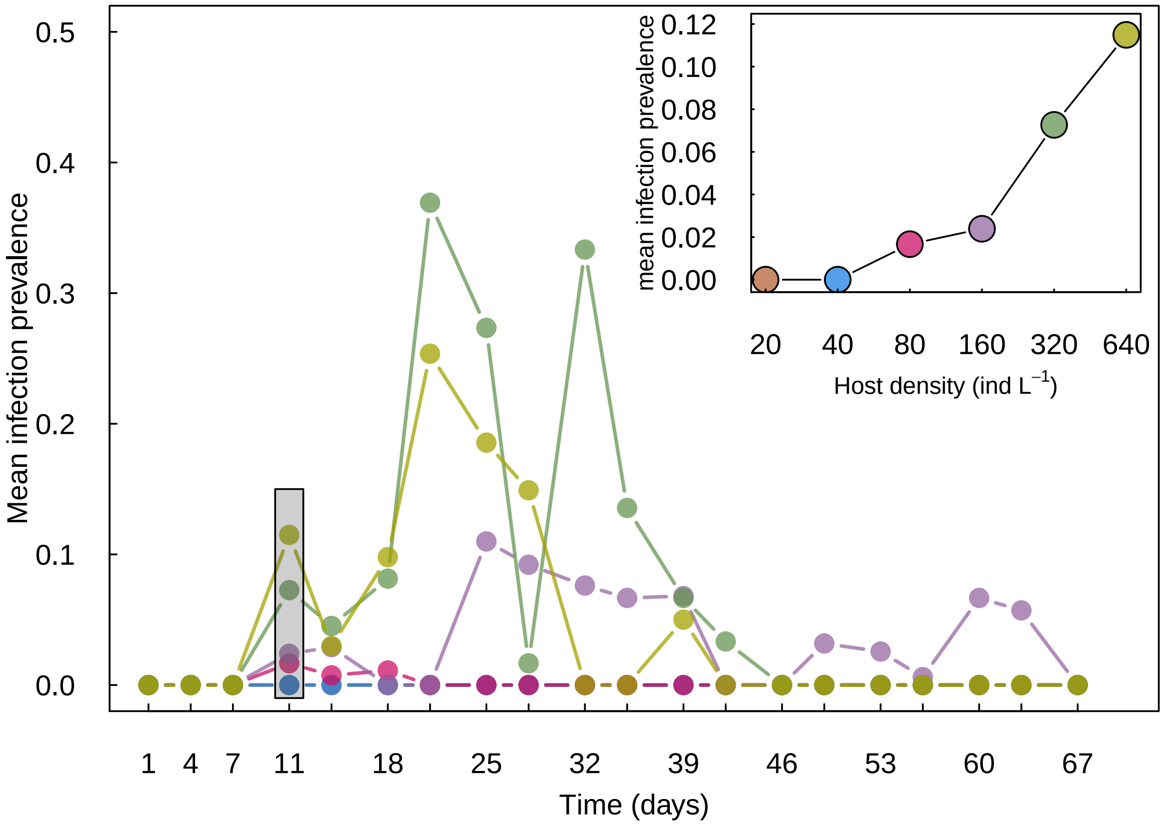

Figure A4: Time-series of experimental infections reveal wave-like infection patterns after pathogen invasion (grey box at day 11). The inset plot shows the monotonic relationship between host density and mean infection prevalence. Colors for host density treatments are conserved from Figures 3 and 4, and are also the same in the inset plot (e.g., orange corresponds to 20 host individuals L^{-1}).

References

[1] Helgen, J. C., 1987 Feeding rate inhibition in crowded *Daphnia pulex*. *Hydrobiologia* **154**, 113–119.

[2] Diekmann, O., Heesterbeek, J. & Metz, J. A., 1990 On the definition and the computation of the basic reproduction ratio r0 in models for infectious diseases in heterogeneous populations. *Journal of mathematical biology* **28**, 365–382.

[3] Bani-Yaghoub, M., Gautam, R., Shuai, Z., van den Driessche, P. & Ivanek, R., 2012 Reproduction numbers for infections with free-living pathogens growing in the environment. *Journal of biological dynamics* **6**, 923–940.

[4] Civitello, D. J., Pearsall, S., Duffy, M. A. & Hall, S. R., 2013 Parasite consumption and host interference can inhibit disease spread in dense populations. *Ecology letters* **16**, 626–634.
